# The University of Salerno’s Model for Seasonal Influenza Vaccinations in the Workplace

**DOI:** 10.3390/vaccines14040359

**Published:** 2026-04-17

**Authors:** Francesco De Caro, Nadia Pecoraro, Francesca Malatesta, Simona Caruccio, Federico Della Rocca, Alessandra Mea, Matteo Tomeo, Raffaele De Caro, Giuseppina Cersosimo, Arcangelo Saggese Tozzi, Anna Luisa Caiazzo, Giovanni Boccia, Emanuela Santoro, Mario Capunzo, Giuseppina Moccia

**Affiliations:** 1 Public Health Laboratory for the Analysis of Community Health Needs, Department of Medicine and Surgery, University of Salerno, Baronissi Campus, 84081 Baronissi, Italy; fdecaro@unisa.it (F.D.C.); fmalatesta@unisa.it (F.M.); 2 Department of Medicine and Surgery, University of Salerno, Baronissi Campus, 84081 Baronissi, Italy; scaruccio@unisa.it (S.C.); fdellarocca@unisa.it (F.D.R.); amea@unisa.it (A.M.); mtomeo@unisa.it (M.T.); r.decaro8@studenti.unisa.it (R.D.C.); gboccia@unisa.it (G.B.); esantoro@unisa.it (E.S.); mcapunzo@unisa.it (M.C.); 3 Department of Political and Social Studies, University of Salerno, 84084 Fisciano, Italy; gcersosi@unisa.it; 4 ASL Salerno, 84091 Salerno, Italy; a.saggesetozzi@aslsalerno.it (A.S.T.); a.caiazzo@aslsalerno.it (A.L.C.)

**Keywords:** seasonal influenza vaccination, vaccination in the workplace, confidence, university vaccination campaigns, Salerno’s model, public health strategies

## Abstract

**Background**: During the flu season, there is an increase in absenteeism due to illness, a drop in productivity, and a greater risk of the virus spreading among workers. Thus, the Italian Ministry of Health recommends vaccination for essential service workers. The University of Salerno, in collaboration with the local health authority of Salerno, offers free vaccination to its employees. **Methods**: A public health methodology for seasonal influenza vaccination in the workplace is presented—specifically in the university setting—with the aim of identifying individual, contextual, and organizational elements of the model that have promoted vaccination uptake. An ad hoc questionnaire was used (October–December 2025) to survey 399 academic employees, investigating seasonal influenza vaccination in the following aspects: recent personal experiences, motivations, vaccination experiences at university, sources of information, considerations regarding national and local vaccination campaigns, and level of vaccine confidence (VCI). **Results**: Seasonal influenza vaccination at the University is appreciated for its compatibility with working hours (66.1%), the availability of a platform that allows flexible booking (56.9%), the perception of safety in the environment (31.6%), the fact that the vaccine is free (17.4%), and the involvement of office/laboratory colleagues (5%). Participants appreciate the model and would apply it to other vaccinations at the University and in other institutional settings. A significant relationship (F = 7.24; df = 1; *p* < 0.05) exists between confidence in the vaccine and the sense of security experienced when receiving the vaccine in the workplace. Data analysis was performed using the IBM SPSS v.28 software. **Conclusions**: The model proposed can be applied to other institutional contexts, simplifying and facilitating access to vaccines by implementing vaccination campaigns tailored to specific work environments.

## 1. Introduction

Each year, influenza affects approximately 5–15% of the world’s population, posing a serious threat to global public health, particularly for older adults and those with chronic conditions, and leading to severe illness and death [1]. It also constitutes a significant source of direct and indirect costs associated with the implementation of control measures and the management of cases and complications of the disease [2].

In accordance with World Health Assembly resolution 56.19, all Member States have committed to increasing immunization efforts to achieve 75% influenza vaccination coverage for older people and people with chronic conditions [3]. The World Health Organization (WHO) has adopted the Global Strategy on Influenza (2019–2030), the objective of which is to prevent and control influenza through the development of tools such as vaccines, antivirals, and non-pharmaceutical interventions [4,5,6,7].

From 2018 to 2023, there was a growing trend in the issuance of national policies and recommendations by Member States to promote seasonal vaccination, leading to increased uptake and higher vaccination coverage [8]. Despite the overall increase, most Member States have not reached the recommended target. To support countries in reaching this global target, the World Health Organization (WHO) has developed a package of quantitative and qualitative resources, namely, the guidebook Behavioural and Social Drivers of Influenza Vaccination: Tools and Practical Guidance for Achieving High Uptake [9,10].

Throughout the 2024–2025 season, the estimated number of cases of flu-like illness, relative to the total Italian population, was approximately 16,129,000 [11]; it was particularly severe, with a worrying increase in hospital admissions, including among children, the elderly, and immunocompromised adults. In Italy, the current National Immunization Plan (PNI 2023–2025), the National Vaccine Prevention Plan (PNPV) 2023–2025, and the annual ministerial circulars have reiterated the minimum vaccination coverage target of 75% and the optimal target of 95% for individuals over 65 years of age and high-risk groups [12,13,14]. However, although Italian data on seasonal influenza vaccination coverage in the general population for the 2024/2025 season, updated as of 29 August 2025, show a slight increase (19.6%) compared to the previous season (18.9%), there was a decrease in the age group over 65 years [14].

The literature suggests that individual, social, and contextual variables are important factors in seasonal influenza vaccination [15,16,17,18], as they affect flu vaccine uptake, refusal, or hesitancy [19,20,21,22,23,24,25,26,27,28].

Research on vaccine adherence has identified several individual determinants, including gender, level of education [25], age, knowledge of the infection, information-seeking behavior [18,29], beliefs and representations about health and prevention [30,31,32,33], and levels of anxiety and stress [17,34,35]. Contextual determinants encompass socioeconomic status, cultural background, religion, geographical barriers, access to vaccinations (such as organizational management, on-site vaccinations, and free vaccine provision), social acceptance, communication, and media, including information campaigns [36,37]. The primary factors underlying vaccine hesitancy include a low perception of disease risk, concerns regarding vaccine safety, lack of confidence in vaccine effectiveness, and limited awareness of vaccine existence and availability [36,38].

Betsch’s model, named the “5C Scale” [39], measures the antecedents that determine flu vaccination behavior, identifying five key dimensions: confidence, complacency [38], constraints, calculation and, finally, collective responsibility [40,41]. The “7C” model adds two more factors: conformity and conspiracy [42].

Specifically, confidence is a significant factor associated with vaccine uptake and can be considered the result of a process that simultaneously involves both individual aspects related to knowledge and experience, as well as contextual factors such as trust in the vaccine (as a product), in healthcare professionals (as providers), and in decision-makers (as policy-makers) [43], in addition to vaccination campaigns and the sources of information chosen by individuals [28,44,45,46]. Conversely, mistrust can be influenced by negative experiences, mistrust of policy-makers, or particular religious or philosophical beliefs [42,47].

This overview shows that adherence to seasonal influenza vaccination and vaccination campaigns requires multilevel actions. The success of vaccination programs depends on a complex combination of logistical, social, and structural factors [48,49,50]; for example, SAGE (Strategic Advisory Group of Experts on Immunization) has emphasized the importance of activating institutional and organizational systems at local and global levels to promote vaccination uptake [21,22,25,50,51]. The ecological model [52,53], applied to public health research and prevention interventions [50], has demonstrated the complex nature of health action, highlighting the need to understand the interactions between individual and contextual factors, including sociocultural and organizational aspects.

### Vaccination in the Workplace

With the arrival of flu season, companies face more sick leave and lower productivity [54]. Indeed, there is a greater risk of the virus spreading among workers [55,56]. In this context, seasonal influenza vaccination is a strategic health prevention strategy. It protects individual employees and collective well-being, prevents the spread of the virus, and reduces absenteeism, ultimately promoting the operational continuity of the organization.

The Italian Ministry of Health, in accordance with WHO international guidelines, recommends vaccination for essential service workers (healthcare workers, law enforcement officers, teachers, etc.) through its annual circulars on the seasonal influenza vaccination campaign [57]. The literature shows that, in educational settings such as universities, contact with the public increases the rate of influenza infection among staff by an average of about 35% [54].

In Italy, vaccination in the workplace is regulated by occupational health and safety regulations (Legislative Decree 81/2008) [58], health legislation and, in specific cases, extraordinary measures such as those implemented in response to the COVID-19 pandemic, such as the National Protocol for Vaccination in the Workplace and the update of the Health and Safety Protocols [59] (National Protocol of 6 April 2021; “Interim Guidelines”—Joint Ministerial Circular, INAIL, Regions of 12 April 2021). Within the context of public health activities, such regulations have enabled the creation of special vaccination points in the workplace through coordination between the company, the occupational physician, and the local health authority/INAIL. Vaccination is voluntary unless there are specific regulatory requirements, preserving the privacy, safety, and dignity of workers in compliance with current regulations on security and data processing (Provision of 13 May 2021; EU Regulation 2016/679-GDPR and the Privacy Code-Legislative Decree 196/2003) [60].

In general, although it is not regulated by a single national standard, many companies, in consultation with a competent physician, offer vaccination in the workplace during working hours, considering it an integral part of health and safety promotion measures.

Currently, there is a lack of data in the literature on the risk of transmission and the impact of influenza in university settings. Additionally, there are no research studies or public health intervention strategies targeting influenza vaccination campaigns in non-healthcare university settings. In recent years, some Italian universities—such as those in Salerno, Catania, Messina, and Milan (UNIMI)—have offered vaccinations to their staff. In addition, the universities of Catanzaro and Parma have begun vaccinating students in medical and health-related degree programs.

The University of Salerno, with its Department of Medicine, Surgery, and Dentistry and the Territorial Health Service, began seasonal influenza vaccination campaigns for teaching and technical/administrative staff in 2022 and has continued since [51]. The number of vaccinated staff has increased progressively over the years: 244 in 2022, 294 in 2023, 356 in 2024, and 399 in 2025. This increasing number corresponds to the number of people added to those already in a participant membership database starting from the first year of administration.

In recent years, the campaigns have been organized by the University’s occupational physicians and the Public Health Laboratory for the Analysis of Community Health Needs, in agreement with the Salerno Local Health Authority and supported by the School of Specialization in Hygiene and Preventive Medicine. Vaccinations were administered at occupational physicians’ clinics, where staff attend scheduled medical examinations. In 2021, the University of Salerno had already started vaccinating staff against COVID-19 at the Vaccination Center of the University Hospital “San Giovanni di Dio e Ruggi d’Aragona” (Salerno, Italy) [51].

The primary aim of this study is to present a public health methodology for seasonal influenza vaccination in the workplace—specifically, in a university setting—with the goal of developing a replicable vaccination model based on an analysis of the work environment and the specific target population.

The goal is to identify whether individual, contextual, and organizational elements of the model have encouraged vaccination uptake.

## 2. Materials and Methods

### 2.1. Setting Procedure

A public health intervention was implemented as part of a national and local seasonal influenza vaccination program launched in Italy between October and December of 2025, with the aim of developing and implementing a specific vaccination strategy for staff at the University of Salerno—the main public university in the province of Salerno (Campania), which attracts students from other provinces and neighboring regions. During the period covered by this study, the University had an average of between 35,000 and 40,000 students and 2247 members of staff.

The ecological matrix model [52,53], originally designed in the social and community sciences and previously used for vaccination against Herpes Zoster [50], involves five levels of analysis/intervention. The macro-systemic level focuses on national and local policies. In Italy, there is the National Vaccination Prevention Plan 2023–2025 (PNPV) and the Vaccination Calendar, which is renewed annually. The community level is represented by the drafting of the application protocol, based on ministerial recommendations, which provided for partnership between the Salerno Local Health Authority and the “Public Health Laboratory for the Analysis of Community Health Needs” of the Department of Medicine, Surgery, and Dentistry (“Scuola Medica Salernitana”) of the University of Salerno. At this stage, the Rector, in agreement with the Local Health Authority, requests the activation of vaccination at the University on an annual basis.

In terms of organization, the intervention involved the following series of actions:

(a) Creation of an interface platform for employees to simplify scheduling and provide information on the University’s vaccination campaign;
(b) Creation of a specially designed brochure providing information on the flu situation, who should be vaccinated, how the vaccine is administered, possible adverse reactions, and how to sign up and collect information;
(c) Pre-registration request for seasonal influenza vaccination via email, to express intention to be vaccinated and define an indicative number of vaccines to be requested from the local health authority, avoiding waste of vaccine doses and simplifying the planning of activities dedicated to vaccine administration;
(d) Planning of the vaccination campaign with a schedule and times dedicated to the administration of each vaccine dose. The time scheduled between vaccinations is useful to allow the administering doctor sufficient time to interact with the patient. The schedule allows workers to organize their working day;
(e) Employee booking on a digital platform, where they can choose the date and time of vaccination. At this stage, the vaccination information and details of the vaccination location are sent again to simplify access to vaccination.

At the microsystem and individual levels, the intervention focused on communication within a physical and preventive context already familiar to employees, as well as understanding the individual, contextual (cultural), and organizational factors relevant to the implementation of the University’s corporate vaccination model. The individual variables we considered are sociodemographic variables, previous history of seasonal influenza vaccination, and confidence in the vaccine (e.g., in terms of as a representation of flu, as a reflection of the virus, the effectiveness of the vaccine, or fear of the vaccine and its side effects). The contextual variables we considered included a culture of belonging, which is conveyed by professional roles, communication, and media (information campaigns). Finally, as organizational variables, we consider the context of vaccination and experience with vaccination in the workplace.

The exploratory observational study presented here was conducted within the scope of a larger research project, of which only a part is presented.

### 2.2. Participants

A total of 399 employees at the University of Salerno took part in the seasonal anti-seasonal influenza vaccination campaign. The workers were teaching staff, administrative employees, Ph.D. students, research fellows, and postdoctoral researchers. This is a convenience sample, as only those who agreed to be vaccinated participated in this research and completed the survey instruments. The survey included 21.55% of the University’s teachers (N = 1132; *n* = 244), 21% of the administrative employees (N = 645; *n* = 135), and 3.5% of the Ph.D. students, postdoctoral researchers, and research fellows (N = 579; *n* = 20).

### 2.3. Tools

The exploratory survey was conducted using an ad hoc questionnaire created based on a review of the literature and the expertise of the specialists involved in this study. The questionnaire was uploaded to the Google Forms platform via a QR code. Participants were asked to complete the questionnaire during the vaccination phase. It includes the following sections:

(1) An informed consent and privacy policy form, which informed participants of the purpose of this research and obtained their informed consent to the use of their data in an anonymous and aggregated form, in accordance with the European Union’s General Data Protection Regulation. This section includes information about the vaccine to be administered (injection site, batch number, expiration date, route of administration, and healthcare professional details).
(2) Individual data, including age, demographic information, education level, and occupation.
(3) An ad hoc scale was created for the survey. The scale consists of 20 items with multiple-choice questions, and has not been previously validated. The dimensions explored were as follows:
  • Experience of seasonal influenza vaccination in recent years and motivation for seasonal influenza vaccination;
  • Experience of vaccination in the workplace, University of Salerno, and reflection on the proposed vaccination model;
  • Sources of information on seasonal influenza vaccines and considerations of national and local vaccination campaigns.

Only the results of the items that provided useful and significant information were included in the findings.

(4) The level of confidence in the flu vaccine (Vaccine Confidence Index—VCI) [44]. The Vaccine Confidence Index (VCI) is derived from eight Likert-type statements included in the staff questionnaire, with which participants were asked to declare their agreement or disagreement. The statements are as follows: A1: Flu is a serious illness. A2: Flu vaccine is effective. A3: Healthcare workers must get vaccinated. A4: By getting vaccinated, I protect people close to me from flu. B1: It is better to get flu than the vaccination. B2: Flu vaccines have serious side effects. B3: The vaccine can cause flu. B4: Opposed to vaccination. The level of agreement or disagreement was scored as follows, with each item predicting responses from 1 to 4 (4 = totally agree; 1 = totally disagree). For the first four statements (A1–A4), the higher the Likert score, the better the propensity toward vaccines; meanwhile, for the second four (B1–B4), the higher the Likert score, the lower the propensity.

The survey was designed to take approximately 20 min to complete.

### 2.4. Statistical Analysis

The data relating to the second (sociodemographic) and third sections of the questionnaire were subjected to descriptive statistical analysis, including analysis of absolute frequencies and percentages, and mean and standard deviation for the age variable. For some items, the percentage was based on multiple responses. A Chi-square analysis was conducted to cross-reference the responses to the items, while an analysis of variance (ANOVA) was performed to cross-reference the frequencies with age (only results with a *p*-value < 0.005 were considered).

The VCI was calculated as follows:

(1) VCI = [(A1 + A2 + A3 + A4)/4]/[(B1 + B2 + B3 + B4)/4]

where A1, A2, A3, and A4 are the scores for the first four statements, while B1, B2, B3, and B4 are the scores for the second four. The percentile indices of the VCI scores were also identified. The VCI scale does not have a cutoff point; the higher the scores, the greater the confidence index. The normality of the data distribution was verified using Kolmogorov–Smirnov and Shapiro–Wilk tests. The relationship between the VCI scores and the responses to the previous sections was assessed using analysis of variance (ANOVA). The VCI was analyzed in relation to age using Spearman’s rho. Only results with a *p*-value < 0.005 were considered. Data analysis was performed using the IBM SPSS v.28 software (IBM^®^SPSS^®^, Bologna, Italy).

## 3. Results

### 3.1. Sample Description

A total of 399 University of Salerno employees have joined the seasonal influenza vaccination campaign. They account for 17% of the working population (N = 2347) and include the University’s teaching and administrative employee population, Ph.D. students, postdoctoral researchers, and research fellows; these individuals took part in the survey voluntarily and anonymously.

Within the sample under consideration, women account for 38.6%, while men account for 61.4% (F = 38.6%; M = 61.4%; mean age = 51.8, SD = 9.2). The mean age is 51.8 (SD = 9.2); women had a mean age of 50 years (SD = 8.8) and that for men was 53 years (SD = 9.2).

In relation to the type of working roles in the university, 33.6% were administrative employees, 61.4% were teachers, and 5% were Ph.D. students/postdoctoral researchers/research fellows.

Regarding profession and gender, participants are distributed differently (Chi-square = 6.621; DF = 2; *p* = 0.036). In the case of Ph.D. students/postdoctoral researchers/research fellows, the percentage is higher for women (M = 35.3%; F = 64.7%); meanwhile, for the teaching profession, we observed an opposite trend (M = 65.4%; F = 34.6%). Technical and administrative employees are distributed with a higher percentage for men (M = 58.8%; F = 41.2%).

Teachers (mean age = 52.5; SD = 8.6) and employees (mean age = 53.1; SD = 8.1) are significantly older (F = 37.7; DF = 2; *p* < 0.001) than Ph.D. students/postdoctoral researchers/research fellows (mean age = 34.8; SD = 5.6).

The level of schooling was as follows: 8%, secondary school diploma; 21.5%, university degree; and 70.5%, postgraduate training (Table 1).

### 3.2. Experience of Seasonal Influenza Vaccination and Motivation for Seasonal Influenza Vaccination

With respect to the flu vaccine, 9.5% have been vaccinated for more than 10 years, 24.3% for 5 to 10 years, and 56.7% for 1 to 5 years, while 9.5% are being vaccinated for the first time.

With regard to the benefits of seasonal influenza vaccination, 7.5% say they have not experienced any benefits, while 27.8% have been ill less often, and 64.7% have had less severe symptoms (Table 2).

**Figure 1:**
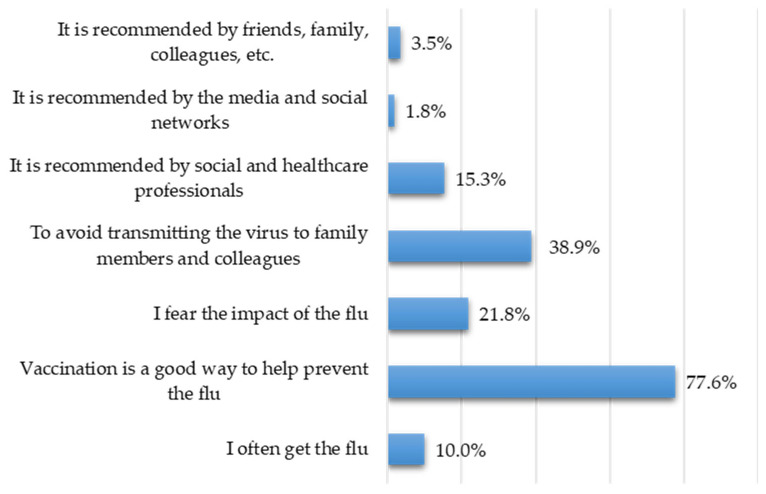
Frequencies in multiple-response set regarding vaccination information.

Participants cite prevention (*n* responses = 657) as their main motivation for vaccination (*n* = 302; 77.6%), followed by protecting family members and colleagues from infection (*n* = 151; 38.9%), and fear of the effects of flu (*n* = 85; 21.8%) (Figure 1). In general, the role of social and healthcare workers (*n* = 53; 15.3%) influences motivation, unlike the advice of family members, friends, or colleagues (*n* = 14; 3.5%) or the media (*n* = 7; 1.8%). The experience of becoming ill often (*n* = 39; 10%) should not be underestimated.

Men are more likely than women to get vaccinated to avoid infecting family members, friends, and colleagues (F = 45.5%; M = 54.5%) (Chi-square = 4.61; DF = 1; *p* = 0.03). Women are more likely than men to cite the need to get vaccinated because they often experience sickness (F = 41.2%; M = 58.8%) (Chi-square = 6.7; DF = 1; *p* = 0.002). Researchers/teachers are the most likely to consider vaccination a useful preventive measure (Chi-square = 8.506; DF = 2; *p* = 0.014).

### 3.3. Experience of Vaccination at the University

**Figure 2:**
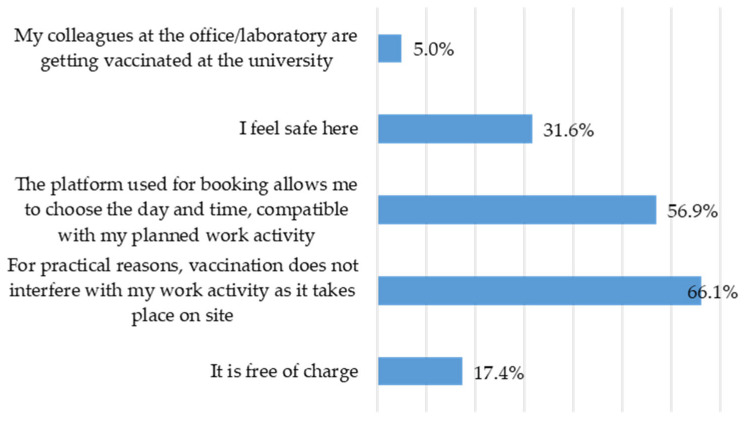
Frequencies in multiple-response set for motivating reasons for seasonal influenza vaccination in University.

There are various reasons behind the decision to get seasonal influenza vaccinations. The main reason is convenience (*n* responses = 688): the vaccination does not interfere with working hours (*n* = 257; 66.1%). The second reason concerns the methodology used by the platform, which allows users to choose the day and time that best suits their work schedule (*n* = 221; 56.9%). The third reason is the perception of safety in the environment (*n* = 123; 31.6%), followed by the fact that the vaccine is free (*n* = 68; 17.4%) and then the involvement of office/laboratory colleagues (*n* = 19; 5%) (Figure 2).

Participants have been involved in this vaccination program since its beginning in 2021 in 49.6% of cases, for 20.4% it is their first time, 15.3% have been vaccinated at least twice since 2021, and 14.7% at least once. The model of vaccination in the workplace encouraged 99% of participants to choose to be vaccinated, and it would be considered useful for other types of vaccinations to be carried out at the University for 97.3% of the participants. The remaining 2.7% do not consider it useful for this purpose (Table 3).

Participants believe it would be useful to extend the model to other institutions/administrations and universities, as it is easy to schedule vaccinations (*n* = 251; 74.6%) and reconciles family/work needs (*n* = 207; 61.4%); in contrast, 0.3% (*n* = 1) do not consider it a useful approach (Table 4).

### 3.4. Sources of Information Used for Vaccination

**Figure 3:**
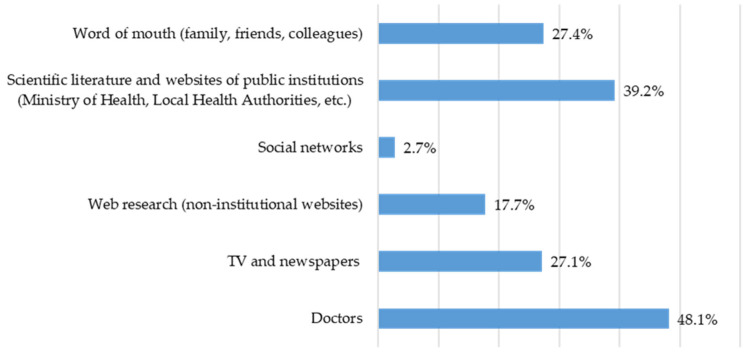
Frequencies in multiple-response set for sources of information used on vaccines and vaccination, in addition to those of the University.

In this section, information was requested regarding the sources of information used in order to decide to get the seasonal influenza vaccine. The sources of information used on the vaccine and vaccination, in addition to those provided by the University, were as follows (*n* responses = 550): family doctor (*n* = 187; 48%), the scientific literature and websites of public institutions (Ministry of Health, Local Health Authority, etc.) (*n* = 152; 39.2%), word of mouth (*n* = 107; 27.4%), TV and newspapers (*n* = 105; 27.1%), web research (non-institutional websites) (*n* = 69; 17.7%), and social networks (*n* = 11; 2.7%) (Figure 3).

**Figure 4:**
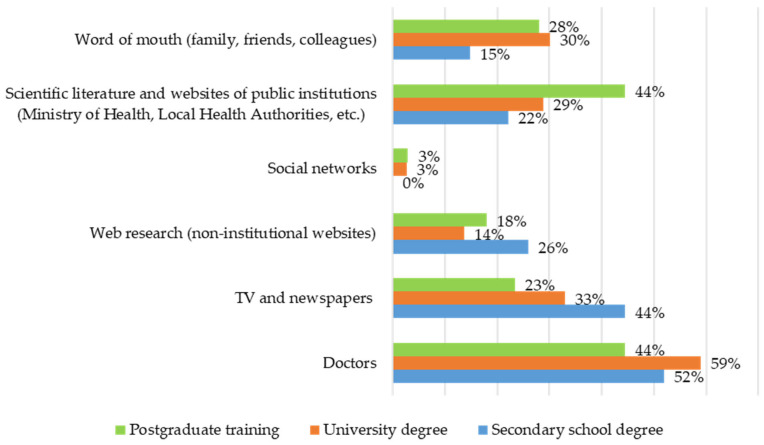
Frequencies in multiple-response set of “Sources of information used for vaccination by training groups”.

In comparison to others, participants with postgraduate training make greater use of the scientific literature (44.4%). Those with secondary training, in comparison to others, use newspapers, television, and research on non-institutional websites more frequently (25.9%). Family doctors are the most popular source of information for those with a university degree (59%), compared to others (Figure 4).

**Figure 5:**
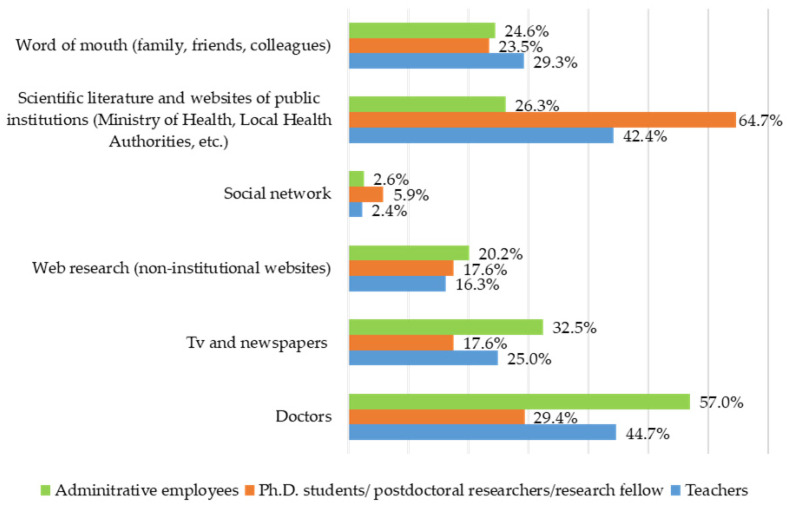
Frequencies in multiple-response set of “Sources of information used for vaccination for professional groups”.

Technical and administrative employees mainly use information from their family doctor (57%), TV and newspapers (26.3%), and non-institutional websites (20.20%). Ph.D. students/postdoctoral researchers/research fellows primarily use the scientific literature and institutional websites (64.7%) (Figure 5).

Regarding the Italian government’s seasonal influenza vaccine information/awareness campaigns, 30.1% express satisfaction, the same percentage (30.1%) are dissatisfied, and the remaining 39.8% are neither satisfied nor dissatisfied. The perception of satisfaction with the University’s information/awareness campaign is different: 76.6% are satisfied, 4.5% are dissatisfied, and the remaining 18.9% are neither satisfied nor dissatisfied. The 68.4% believe it would be useful to receive more information about seasonal influenza and seasonal influenza vaccination at the University, while the remainder (31.6%) do not consider it necessary (Table 5).

### 3.5. Vaccine Confidence Index

The result of the VCI test is X = 2.79 (SD = 0.83; median = 2.80; range: 0.92–4) with the value around the 50th percentile; more precisely, between the 25th (value = 2.14) and 50th (value = 2.80) percentiles. The VCI is not distributed normally.

The vaccine adherence score differs significantly (F = 6.26; DF = 1; *p* = 0.013) between men (X = 2.88, SD = 0.84) and women (X = 2.65, SD = 0.83), with greater adherence among men.

Profession also appeared to be a determinant (F = 18.96; df = 2; *p* < 0.001), with vaccine confidence being higher among Ph.D. students/postdoctoral researchers/research fellows (mean = 3.13; SD = 0.76), lower among teachers (mean = 2.96; SD = 0.79), and even lower among technical and administrative employees (X = 2.42; SD = 0.80).

There was also a significant relationship with educational attainment (F= 4.88; DF = 2; *p* = 0.008). A higher level of education corresponds to a higher vaccination compliance rate: secondary school degree (mean = 2.48; SD =0.89), university degree (mean = 2.62; SD = 0.82), and postgraduate training (mean = 2.88; SD = 0.82).

Concerning the relationship between motivation to get vaccinated and the VCI, confidence is related to the idea that vaccination is an effective means of prevention (F = 14.98; DF = 1; *p* < 0.001) and is carried out on the recommendation of friends, colleagues, and family members (F = 3.75; DF = 1; *p* = 0.021).

Regarding the relationship between VCI and vaccination information/experience, there is a relationship between vaccine flu confidence and previous vaccination adherence (F = 2.639; DF = 4; *p* < 0.034).

Those who have been vaccinated for more years have a higher score on the VCI: “For more than 10 years” (mean = 3.06; SD = 0.90), “5 to 10 years” (mean = 2.86; SD = 0.83), “1 to 5 years” (mean = 2.76; SD = 0.79), and “this is the first time I have been vaccinated” (mean = 2.49; SD = 0.94).

Confidence in vaccination is significantly related to the positive health effects observed after seasonal influenza vaccination (F = 2.85; DF = 3; *p* = 0.37), with greater confidence observed in those who had milder symptoms after vaccination (mean = 2.80; SD = 0.76) or who became infected less often (mean = 2.84; SD = 0.85).

Regarding the University vaccination experience, there is a significant relationship between VCI and the sense of security of vaccination at the University (F = 7.24; DF = 1; *p* = 0.007). Furthermore, there is a relationship between VCI and the perception of the usefulness of the vaccination model proposed by the University (F = 3.95; DF = 1; *p* = 0.047).

Those who have more confidence in the vaccine say that the proposed model could also be extended to other vaccines in the university context (F = 10.78; DF = 1; *p* = 0.001), and confidence is linked to the possibility of reconciling work and vaccination efforts (F = 7.37; DF = 1; *p* = 0.007).

Regarding the information used, participants with a higher VCI find it useful to be able to use the platform created by the University to book vaccinations for their loved ones with their primary doctor (F = 8.25; DF = 1; *p* = 0.004). Those with a higher VCI are critical of government information campaigns on seasonal influenza (F = 5.12; DF = 2; *p* = 0.004).

Regarding communication, those who obtained more detailed information through channels such as the scientific literature and public institution websites had a higher VCI (F = 7.73; DF = 1; *p* = 0.006).

## 4. Discussion

The main objective of this study was to present a public health methodology for seasonal influenza vaccination in the workplace, identifying the individual, contextual, and organizational factors within the model that contributed to the uptake of vaccination in order to implement workplace vaccination models that are replicable and adaptable to different contexts. The goal is not only to address the need for preventive measures, but also to reduce staff absenteeism and the associated costs, which, in Italy, are around 10% [61]. According to an Italian study, employers can save up to EUR 314 for each employee who agrees to get a flu shot (taking into account the costs incurred by the organization to carry out the vaccination campaign, compared to the savings achieved through reduced sick leave). This is considered an additional advantage for the University, as it has not had to bear the costs of procuring vaccines itself, which are covered by the local healthcare system [62,63,64].

Currently, the percentage of vaccinated staff among the total workforce is still relatively low, at 17%, compared to 19.6% of national vaccination data [2]; however, it helps create an initial barrier against the spread of the virus, both in the workplace and among the general population. The abovementioned two-point difference does not seem significant to us, as the national figure includes vulnerable groups and those over 65—who are only slightly represented in our sample—and, furthermore, it also pertains to the vaccination of healthcare workers.

The results of this study indicate that promoting vaccination campaigns in the workplace, on a voluntary and free basis, facilitates easier access for workers, leading to wider and more timely uptake. This uptake is the result of a multilevel process where various factors jointly affect flu decision outcomes [18,21,25,29,30,35,50,51].

The multilevel ecological model offers a method to identify key factors affecting vaccination in the context of the University’s seasonal influenza vaccination campaign, as also captured by the 5C model [65]. The latter emphasizes confidence as the primary determinant of vaccine compliance, highlighting the roles of the vaccination healthcare system (organizational level) and its staff (microsystemic level) as fundamental.

Although the vaccine confidence index (VCI) data hover around the 50th percentile—indicating that confidence in vaccines is not particularly high—employees believe that the model encourages vaccination. This confirms that confidence, as a “mental attitude of relying on the vaccine,” is not sufficient to determine whether individuals will ultimately choose to get vaccinated. Contextual and organizational factors, such as trust in the vaccinator or other healthcare professionals (the provider) and trust in decision-makers, as well as the sense of security derived from the way in which the organizational context facilitates healthcare practices, are fundamental [25]. In addition, previous vaccination experiences, leading to both a reduction in symptoms and a decrease in the incidence of the virus, were observed to influence vaccine confidence.

Those who experienced the University vaccination model felt a sense of security, linked to familiar places, contexts, and staff. This perception of safety was based on previous experiences of vaccinations carried out at the university, even during the COVID-19 emergency, as well as on their knowledge about the doctor’s competency, the dedication of healthcare and administrative staff to the vaccination campaign, and the clinics where they usually go for scheduled medical examinations.

The participants believe, based on this perceived trust, that the model could be extended to other healthcare contexts, such as other vaccines administered in the workplace. Employees who have been vaccinated for several years at the University have full confidence in the proposed vaccination model and in seasonal influenza vaccination, as they have experienced the positive outcomes of vaccination, such as milder symptoms and lower frequency of illness, creating a positive experience and increased complacency [66,67,68].

They also believe that vaccination is a factor in both individual and collective protection (collective responsibility) [41,65,69] that reduces the risk of disease, consistent with studies conducted on seasonal influenza vaccinations among healthcare workers [70,71].

The organizational level of the model used in this study highlighted the fundamental roles of “constraint” aspects of the 5C model [39,65] in promoting vaccine adherence, including the use of a digital platform to receive information on seasonal influenza vaccination and the choice of times and days to get vaccinated, in accordance with work and logistical needs [6].

The use of the platform made it possible to provide employees with a clear reminder of the date, time, and place of vaccination when booking [72]. Furthermore, providing an information brochure with clear and effective information on the epidemiological situation and the type of vaccine during the pre-registration phase made it possible to positively affect flu employees’ decision-making [73], avoiding wasted vaccine doses and leading to a responsible and voluntary choice.

Information is a crucial variable [70], and communication campaigns on prevention can be counterproductive if not adapted to literacy levels [50,71,74,75]. A mismatch can alter individuals’ perception of severity and vulnerability to disease, thus influencing the calculation of the risk/benefit ratio and consequently potentially increasing vaccine hesitancy [23,76,77,78,79,80,81,82,83]. Employees who received information about the virus and vaccination from the University, both before signing up and at the time of vaccination, are satisfied with the University’s information and awareness campaign. However, these employees remain skeptical about national information campaigns.

Despite this, workers still feel the need to receive further information, which leads us to hypothesize, on the one hand, that there is a need to continue to provide in-depth information to overcome the skepticism that stems from misinformation. On the other hand, this could be linked to the specificity of the sample, which has a high level of education and, as the data show, tends to use scientific research as a source of information.

In terms of searching for information to conduct their own risk/benefit analysis of vaccination [39,65], employees mainly used their doctor, the scientific literature, public institution websites, word of mouth, TV, and newspapers. This was influenced by individual factors such as level of education and contextual and cultural factors such as profession.

In the first case, a higher level of education correlates with greater research into information from scientific sources: employees with a higher level of education develop their own knowledge about the vaccine, seek information, read articles, and use institutional websites, which increases their awareness when making decisions [84,85,86]. In terms of profession, technical and administrative employees rely more heavily on communication with the doctor.

Although doctoral students, postdoctoral researchers, and fellows are well informed about the benefits of vaccination, they represent the group with the lowest vaccination rate as a percentage of the sample.

This could result from a reluctance among young people to get vaccinated [62]. Alternatively, because these workers have less contact with the public than faculty members, they might feel less urgency to vaccinate. It is also possible that, due to having fixed-term contracts, they have not developed the habit of getting vaccinated within the university.

The discussion so far suggests that vaccination uptake is not merely a matter of logistical support but, rather, a gradual cultural shift in which awareness-raising and action complement one another and are both essential components for increasing vaccination coverage. Promoting a culture of prevention in the workplace demonstrates the company’s commitment to the health and well-being of its employees. This can only strengthen their sense of belonging and trust in the organization—a fundamental prerequisite for active participation in prevention and health promotion initiatives—and foster confidence in vaccination and the healthcare system.

### Limitations, Advantages, and Future Developments

The vaccination model proposed by the University, in terms of its planning and flexibility with respect to the context and professional needs, represents—in our opinion—a best practice that can be adapted and exported to any work or similar context to increase vaccination uptake.

The implementation of multilevel actions provides a useful framework that can be replicated in different contexts, while also ensuring flexibility for actions that can and must be adapted to the scenarios and collective and individual needs that arise within them [45].

Furthermore, the application of the model, as we have implemented it over the years, promotes the acquisition of elements of trust that increase compliance and adherence to the proposed preventive actions.

Among the limitations of this study is the lack of inclusion of a control group that chose not to be vaccinated; the participants were all proactive about seasonal influenza vaccination, having already pre-registered or been vaccinated at the University in previous years, which limits the generalizability of the data. Furthermore, the survey was conducted exclusively at the University of Salerno; as such, it would be useful to extend this research to other universities.

There are also no other studies on influenza vaccination among non-healthcare university staff, which limits the ability to compare these findings with other data in the literature; as such, this represents a key new area for research development in the field of public health.

It would also be useful to conduct a survey to gather specific feedback from staff regarding the prevention information campaign and improvements to the platform.

Future development could include applying the model to other vaccinations, extending the model to other organizational and similar contexts, and verifying its success in terms of the number of people vaccinated.

Another interesting area of research could focus on trends in the relationship between absenteeism and costs incurred by universities and the social security agency (INPS).

## 5. Conclusions

Vaccination in the workplace can be a useful tool within a public health program, as it helps to address the following points: (A) the requirements set forth in European and national guidelines to increase vaccination coverage; (B) the prevention and protection needs of individual workers, the workplace community, and their families; and (C) overcoming individual and contextual barriers that discourage vaccination uptake or lead to vaccine hesitancy (convenience, cost, and confidence), thereby creating a model of “proximity” to vaccination.

Vaccination is a strategic tool for public health prevention, as it protects both the health of individual employees and the collective well-being of the workforce, while preventing the spread of the virus and its impact on the organization’s costs and productivity. Therefore, it is necessary to implement guidelines for workplace seasonal influenza vaccinations for non-healthcare workers. Workplace vaccination requires intervention models that are contextualized and tailored to specific target groups. These interventions must be designed and tailored based on (a) the context (physical environment, organizational structure, human resources, etc.); (b) the target audience; (c) the type of information and communication created specifically for this purpose, possibly including data that highlight the decline in flu cases over the years at the facility where the vaccination campaign is being conducted; and (d) specific training for healthcare workers involved in the new vaccination models to increase vaccination uptake.

In summary, there is a need for a public health system that includes more accommodating and diverse care settings, where physical barriers are removed and investments are directed toward care that is closer to patients and not confined to hospitals or health centers. In this case, it is conceivable that the responsible institutions could implement vaccination through mobile models in the workplace to create ad hoc vaccination campaigns tailored to the individual work setting, thus improving access to vaccines and increasing vaccination uptake.

## Figures and Tables

**Table 1:** Sociodemographic variables.

Main Categories	Variables		*n*/N (399)	(%)
Sociodemographic	Gender	Men	244	61.4%
		Women	155	38.6%
	Work	Employee	135	33.6%
		Teacher	244	61.4%
		Ph.D. student/postdoctoral researcher/research fellow	20	5%
	Level of schooling	Secondary school diploma	34	8.0%
		University degree	84	21.0%
		Postgraduate training	281	70.5%

**Table 2:** Frequencies of answers to items on seasonal influenza vaccination information.

Item on Information About Seasonal Influenza Vaccination		*n*/N (399)	%
How many years people have been getting the flu vaccine	More than 10 years	38	9.5%
	5 to 10 years	97	24.3%
	1 to 5 years	226	56.7%
	This is my first time	38	9.5%
Benefits of flu vaccination	Get the flu less often	111	27.8%
	Experiencing milder flu-like symptoms	251	64.7%
	No benefits	30	7.5%

**Table 3:** Frequencies of answers to items on seasonal influenza vaccination at the University.

Item on Information About Seasonal Influenza Vaccination at the University		*n*/N (399)	%
Participation in the seasonal influenza vaccination program at the University	Yes, always	198	49.6%
	Yes, once	59	14.7%
	Yes, twice	61	15.3%
	This is my first time	81	20.4%
The decision to get the seasonal influenza vaccine is encouraged by the model proposed by the University	Yes	395	99.0%
	No	4	1.0%
Applicability of the seasonal influenza vaccination model to other vaccinations at the university	Yes	388	97.3%
	No	11	2.7%

**Table 4:** Frequencies in multiple-response set for model extensibility.

Variable		*n*/N (459) Responses	Total Count%
Applicability of the vaccination model promoted by the University to other administrations/institutions/universities	Yes, using a dedicated platform makes it easier and quicker to schedule your vaccination	251	74.6%
	Yes, because vaccination in the workplace reconciles work and family needs	207	61.4%
	No, I don’t think it’s a very useful method	1	0.3%

**Table 5:** Frequencies of items on satisfaction with the seasonal influenza vaccination campaign.

Item on Information About Satisfaction with Information Campaigns on the Seasonal Influenza Vaccine		*n*/N (399)	%
Satisfaction with the information/awareness campaigns on the flu vaccine implemented by the Italian government	Satisfied	120	30.1%
	Not satisfied	120	30.1%
	Neither satisfied nor unsatisfied	159	39.8%
Satisfaction with the information and awareness campaigns on the flu vaccine implemented at the University	Satisfied	306	76.6%
	Not satisfied	18	4.5%
	Neither satisfied nor unsatisfied	75	18.9%
Interest in receiving more information about flu and seasonal influenza vaccination from the University	Yes, it would be helpful	273	68.4%
	No, I don’t think it’s necessary	136	31.6%

## Data Availability

Written informed consent was obtained from the subjects in order to publish this paper. The archived data are not public and can be requested by writing to the corresponding author.
